# Mechanism of *Lysobacter enzymogenes* resistance toward fungi induced by fungal-derived signal α-terpinene

**DOI:** 10.1128/aem.01471-25

**Published:** 2025-09-24

**Authors:** Meixue Zhu, Yuying Li, Fang Nan, Jia Wang, Benfeng Gao, Huihui Song, Zeran Bian, Xuejie Wang, Yuxiang Zhu, Yan Wang

**Affiliations:** 1 MOE Key Laboratory of Evolution and Marine Biodiversity, College of Marine Life Sciences, Ocean University of China 506915 , Qingdao, China; 2 Institute of Evolution & Marine Biodiversity, Ocean University of China 12591, Qingdao, China; 3 Laboratory for Marine Ecology and Environmental Science, Qingdao National Laboratory for Marine Science and Technology 474988 , Qingdao, China; Indiana University Bloomington, Bloomington, Indiana, USA

**Keywords:** *Lysobacter enzymogenes *YC36, bacterial-fungal interactions (BFI), heat-stable antifungal factor (HSAF), GluB, biofilm

## Abstract

Bacterial-fungal interactions (BFI) are critical drivers of ecosystem functions, and their molecular mechanisms remain incompletely understood. Chemical signals are crucial mediators of microbial communication, but the signaling mechanisms of terpene compounds secreted by fungi are not fully elucidated. In this study, the environmental bacterium *Lysobacter enzymogenes* YC36 (*Le*YC36) was employed to systematically investigate the regulatory mechanism of α-terpinene, a fungal-derived terpene, on biofilm formation and antifungal activity. Our results indicate that α-terpinene enhances the competitiveness of *Le*YC36 in BFI by promoting the biosynthesis of antifungal effectors, heat-stable antifungal factor (HSAF), and fungal cell wall hydrolase GluB, as well as biofilm formation. Furthermore, we reveal the α-terpinene-mediated signaling pathway in *Le*YC36. *Le*YC36 detects the fungal signal α-terpinene through *Le*CzcS, the sensor histidine kinase of the two-component system *Le*CzcS/*Le*CzcR, which then transmits the information to the regulator *Le*CzcR. The phosphorylated *Le*CzcR directly stimulates the expression of downstream functional genes *Lepks/nrps*_HSAF_ (*Lehsaf*), *LegluB*, and *Lepsl*, which enhance the production of antifungal effectors (HSAF and GluB), improve biofilm formation, and ultimately boost the competitiveness of *Le*YC36 against fungi. This study elucidates the role of α-terpinene in BFI and its regulatory mechanisms, which provides a novel perspective on communication and cooperative adaptation among microorganisms.

**IMPORTANCE:**

The complex interactions between bacteria and fungi play a crucial role in maintaining ecosystem balance and biodiversity. This study elucidates the molecular mechanism by which α-terpinene, as a fungal-derived signaling molecule, regulates biofilm formation and antifungal activity in *Lysobacter enzymogenes* YC36 (*Le*YC36) and preliminarily establishes the signaling regulatory pathway of α-terpinene. These findings advance our understanding of interkingdom signals between bacteria and fungi and provide a theoretical framework for developing biocontrol technologies using microbial signaling molecules.

## INTRODUCTION

Bacteria and fungi, as essential components of the environmental microbiome, are widely distributed across diverse ecosystems, ranging from soil matrices to host-associated environments, where they form dynamic and co-evolving communities (1, 2). Bacteria-fungi interactions (BFI) not only serve as critical drivers of ecosystem functions but also play a pivotal role in maintaining biodiversity and ecological balance (3, 4). These interactions, which may be antagonistic or synergistic, are shaped by shared habitats, chemical signaling, molecular regulation, and genetic exchange (5). Such interactions influence nutrient competition, cooperative behavior, and niche adaptation, ultimately shaping microbial populations and establishing functional networks critical for ecosystem stability (6–10). Research on microbial chemical signal transduction has primarily focused on the effects of compounds secreted by bacteria on other bacteria or fungi (11, 12). In contrast, the influence of fungal-derived compounds on bacterial physiology remains underexplored (13). Recent studies have demonstrated that fungi can produce more than 300 volatile organic compounds, including a diverse array of chemicals, such as terpenes, alcohols, benzenoids, aldehydes, olefins, acids, esters, and ketones (14, 15). α-Terpinene is a natural monoterpenoid compound that is widely found in plants and some fungi and is usually released into the environment as a secondary metabolite (16, 17). Existing studies have shown that α-terpinene, as a cross-kingdom signaling molecule, can regulate the physiological activities of bacteria and play an important role in the interaction between fungi and bacteria (16). However, the specific mechanisms through which α-terpinene induces competition or cooperation between bacteria and fungi remain unclear. Further investigation is required to explore bacterial response strategies mediated by α-terpinene. A deeper understanding of these processes is beneficial to comprehensively evaluate the role of biodiversity and ecological balance in natural environments.

*Lysobacter enzymogenes* has emerged as a promising biocontrol agent due to its remarkable ability to antagonize fungi and Gram-positive bacteria (18). Unlike other environmental bacteria, *L. enzymogenes* is characterized by its unique ecological adaptability and diverse antimicrobial mechanisms, including the production of extracellular enzymes and secondary metabolites, which contribute to its potent antimicrobial activities (19–24). Among these, the heat-stable antifungal factor (HSAF) has garnered significant attention for its potent antifungal activity and novel mode of action (18, 25). However, information regarding how *L. enzymogenes* YC36 (*Le*YC36) responds to fungal-derived signals remains limited. Understanding the mechanisms and regulatory pathways underlying the interactions between *Le*YC36 and fungi is crucial. Such insights are essential for elucidating the dual strategy of “offense” and “defense” employed by *Le*YC36 against fungal pathogens. This study investigated the impact of the fungal-derived signaling molecule α-terpinene on the antifungal effectors and biofilm production of *Le*YC36, from both offensive and defensive perspectives, and clarified the response and regulatory mechanisms of *Le*YC36 to α-terpinene. It will provide a theoretical foundation for uncovering novel aspects of microbial communication and competition.

## RESULTS

### α-Terpinene enhances the antifungal activity of *Le*YC36

**Fig 1:**
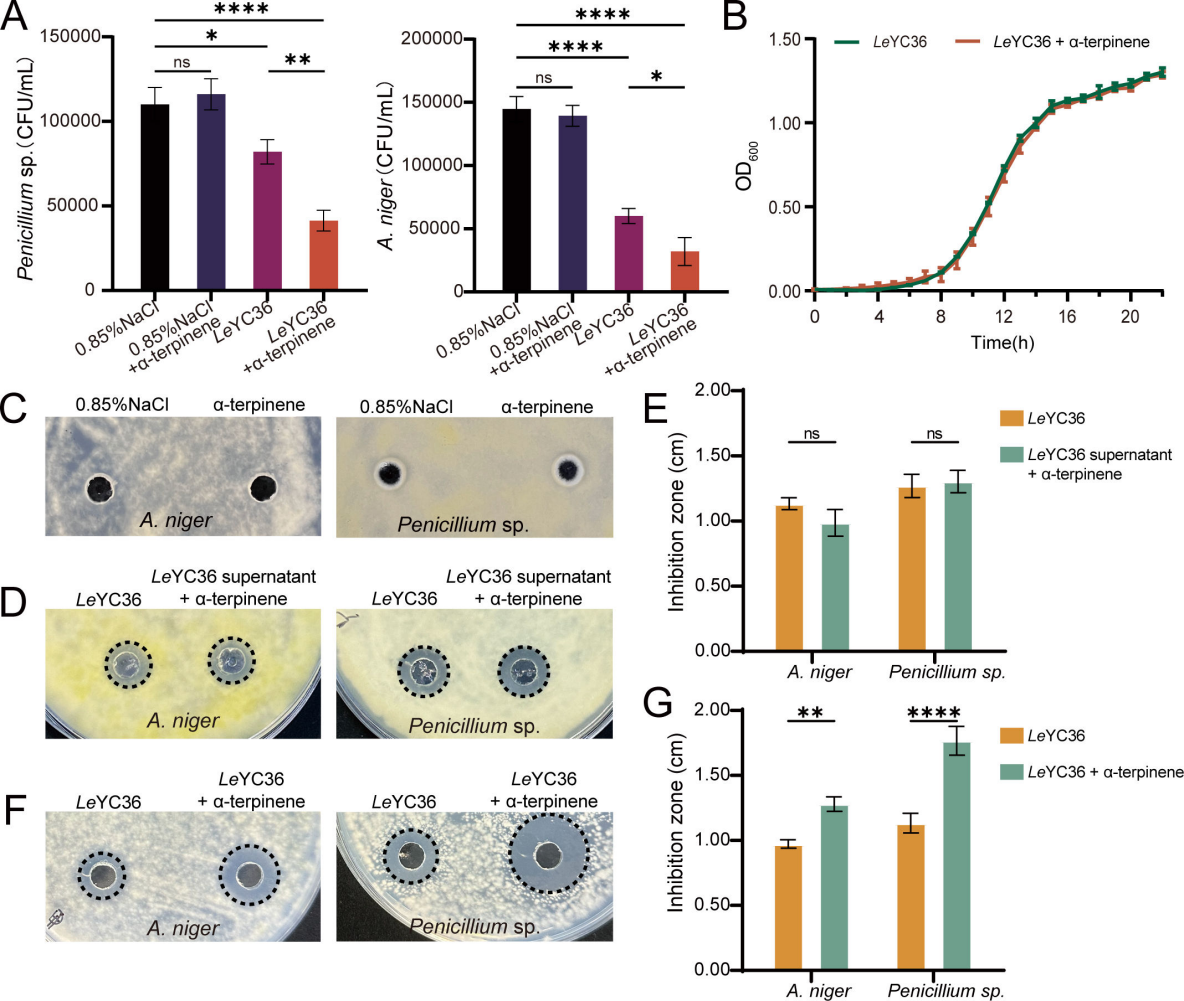
α-Terpinene enhances the inhibitory activity of *Le*YC36 against fungi. (**A**) Fungal CFU assay following *Le*YC36-fungal co-culture. Logarithmic-phase cells of *Le*YC36 and fungal spores were co-cultured for 24 h. The initial fungal concentration was 10^5^ CFU/mL. A 0.1 µM α-terpinene or an equal volume of dimethyl sulfoxide (DMSO) was added during incubation. (**B**) Growth curves of *Le*YC36 with or without α-terpinene. A 0.1 μM α-terpinene or an equal volume of DMSO was added to *Le*YC36 during its growth. (**C**) Effects of α-terpinene on fungi. The right side received saline containing 0.1 μM α-terpinene, while the left side received saline containing an equivalent volume of DMSO. (**D, F**) Agar diffusion assay of *Le*YC36 fermentation supernatant. A 20 µL supernatant was applied to freshly inoculated fungal plates of *Penicillium* sp. or *A. niger*. D *Le*YC36 supernatant + α-terpinene: α-terpinene added post-fermentation to the *Le*YC36 supernatant. F *Le*YC36 + α-terpinene: *Le*YC36 culture supplemented with α-terpinene during growth. The data for the significance of panels D and F are shown in panels E and G. (**E, G**) The inhibition zone diameter results. The inhibition zone diameter was quantified with a vernier caliper to the nearest 0.01 cm. Error bars show the standard deviation of three replicates. *P < 0.05, **P < 0.01, ****P < 0.0001. Data presented as mean  ±  SD.

In the interaction between *L. enzymogenes* and fungi, we found that α-terpinene enhances the antifungal activity of *Le*YC36. We added α-terpinene into the co-culture of *Le*YC36 and fungi, and the results showed that the survival rate (CFU assay) of the fungi *Aspergillus niger* and *Penicillium* sp. in the experimental group (fungi + *Le*YC36 + α-terpinene) was significantly inhibited (Fig. 1A). To determine whether this phenomenon was caused by the effect of α-terpinene on the growth ability of fungi or bacteria, we separately monitored the growth curve of *Le*YC36 and the growth ability of the fungi under α-terpinene conditions. The experimental results showed that α-terpinene did not affect the growth ability of *Le*YC36 (Fig. 1B). As shown in the control group (fungi + 0.85% NaCl + α-terpinene) of Fig. 1A and C, α-terpinene did not inhibit the survival rate and the growth of *A. niger* and *Penicillium* sp. in the absence of *Le*YC36. This suggests that α-terpinene does not enhance the competitive ability of bacteria by promoting bacterial growth or inhibiting fungal growth but rather increases the antifungal ability of *Le*YC36 through more complex regulatory mechanisms. It is known that *L. enzymogenes* produces extracellular enzymes and antifungal factors that inhibit fungal growth. Therefore, we extracted the fermentation supernatants of *Le*YC36 under conditions with or without α-terpinene and evaluated their inhibitory effect on fungal growth. Experimental findings demonstrated that supplementing the *Le*YC36 fermentation supernatant with α-terpinene did not alter fungal inhibition zone diameter (Fig. 1D and E). In contrast, when α-terpinene was added during the *Le*YC36 fermentation process, the resulting fermentation supernatant exhibited an enhanced antifungal effect, as indicated by a significantly larger inhibition zone compared to the control group (Fig. 1F and G). This suggests that α-terpinene may enhance the antifungal capacity of *Le*YC36 by inducing the production of more antifungal substances.

### α-Terpinene promotes the expression of GluB and HSAF to improve the competitiveness of *Le*YC36

**Fig 2:**
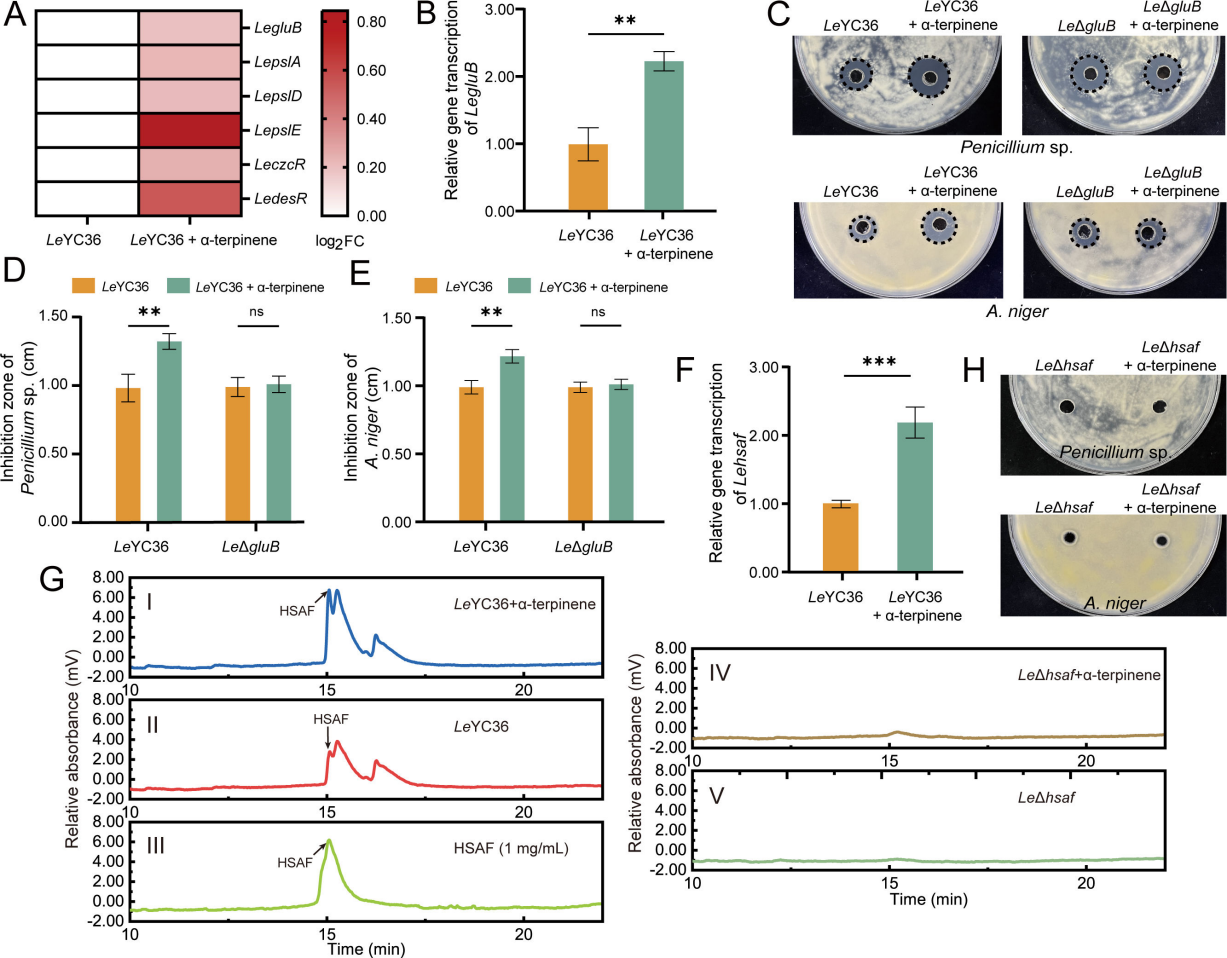
α-Terpinene promotes the production of HSAF and GluB, thereby improving the antifungal activity of *Le*YC36. (**A**) Comparative transcriptome results of upregulated *LegluB*, *LepslA*, *LepslD*, *LepslE, LeczcR,* and *LedesR* genes in *Le*YC36 induced by α-terpinene. (**B**) qPCR result of *LegluB* gene in *Le*YC36 with or without α-terpinene. (**C**) Agar diffusion assay of *Le*Δ*gluB* fermentation supernatant. The data for significance are shown in panels **D** and **E**. (**D, E**) The inhibition zone diameter results of *Le*Δ*gluB*. (**F**) qPCR result of gene *Lehsaf* in *Le*YC36 with or without α-terpinene. (**G**) High-performance liquid chromatography (HPLC) results of HSAF produced by wild-type and mutant strains. III shows HPLC analysis of a 1 mg/mL HSAF standard. Water containing 0.1% formic acid was used for solvent A, and acetonitrile containing 0.1% formic acid was used for solvent B. The flow rate was 1 mL/min, detection wavelength was 320 nm. Gradient (30 min total): 20% solvent B (0 min), to 35% (5 min), to 75% (12 min), to 90% (20 min), to 100% (27 min). (**H**) Agar diffusion assay of *Le*Δ*hsaf* fermentation supernatant. Error bars show the standard deviation of three replicates. **P < 0.01, ***P < 0.001, ns is not significant. Data presented as mean  ±  SD.

Comparative transcriptome analysis revealed that under the induction of α-terpinene, the expression of the *LegluB* gene in *Le*YC36 was significantly upregulated (Fig. 2A). GluB is a β-1,3-glucanase that can hydrolyze fungal cell walls and is closely associated with the antifungal capacity of *L. enzymogenes*. The increased expression level of *LegluB* induced by α-terpinene was verified by quantitative PCR (qPCR) (Fig. 2B). The *Le*Δ*gluB* strain we used was provided by our laboratory. The results showed that the inhibition zone of mutant *Le*Δ*gluB* under α-terpinene induction did not significantly differ from that of the control group, and α-terpinene could no longer promote the antifungal ability of the mutant strain (Fig. 2C through E). This suggests that the *LegluB* gene plays a crucial role in the signaling pathway through which α-terpinene regulates the antifungal activity of *Le*YC36. In addition to extracellular enzymes, *L. enzymogenes* produces a variety of secondary metabolites with antifungal activity, among which HSAF plays a dominant role in antifungal activity. Under α-terpinene conditions, we measured the expression levels of the gene *Lepks/nrps*_HSAF_ (*Lehsaf*), a key synthetic gene of HSAF in *Le*YC36, which encodes a hybrid PKS-NRPS enzyme. As shown in Fig. 2F and G through I, II, α-terpinene significantly upregulated the expression of gene *Lehsaf* and the production of HSAF. After deletion of the *Lehsaf* gene, the mutant strain was unable to produce HSAF (Fig. 2G-IV, V), lost its antifungal activity, and no longer responded to regulation by α-terpinene (Fig. 2H). This indicates that the production of HSAF is a crucial prerequisite for *L. enzymogenes* to enhance its antifungal activity in response to α-terpinene. The results of the above experiments confirm that α-terpinene significantly upregulates the antifungal capacity of *Le*YC36. It is speculated that after sensing the fungal signaling molecule α-terpinene in the environment, *Le*YC36 recognizes the presence of competing fungal species, triggering an increase in the expression of antifungal effectors to maintain its competitive advantage.

### Effect of α-terpinene on the biofilm formation of *Le*YC36

**Fig 3:**
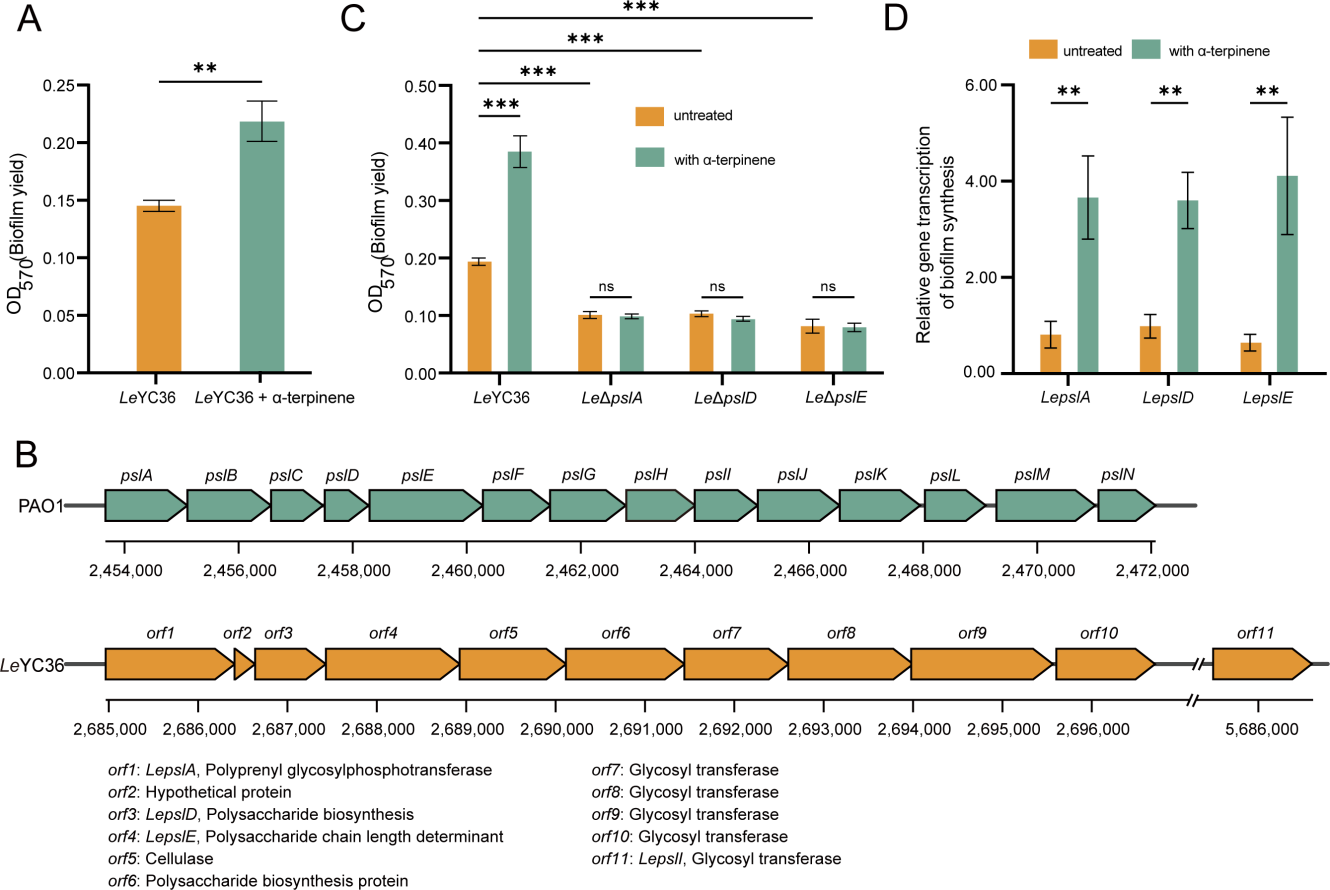
α-Terpinene promotes the biofilm formation of *Le*YC36. (**A**) Biofilm production assay of *Le*YC36 with or without α-terpinene. (**B**) The *psl* gene clusters of *L. enzymogenes* YC36 and *P. aeruginosa* PAO1. (**C**) Biofilm production assay of *Le*Δ*pslA, Le*Δ*pslD*, and *Le*Δ*pslE*. The culture time for each group was the same. During strain growth, 0.1 µM α-terpinene or an equal volume of DMSO (as a control) was added. (**D**) qPCR results of *LepslA*, *LepslD*, and *LepslE* in *Le*YC36 with or without α-terpinene. Error bars show the standard deviation of three replicates. **P < 0.01, ***P < 0.001, ns is not significant. Data presented as mean ± SD.

If HSAF and GluB are considered as the “offensive weapon” of *L. enzymogenes*, then the biofilm serves as the “defensive barrier” during its interaction with fungi. Biofilms can resist fungal toxins, metabolic waste, and other harmful substances produced by fungi, providing bacteria with a relatively stable microenvironment (26). The biofilm plays a crucial role in the interaction between bacteria and fungi, as its formation leads to the accumulation of bacterial virulence factors, ultimately resulting in the death of fungal cells (27). Therefore, we examined whether α-terpinene affects the biofilm expression in *Le*YC36. The results showed that the biofilm production of *Le*YC36 was significantly increased upon induction with 0.1 µM α-terpinene (Fig. 3A). This suggests that the promotion effect of α-terpinene on the competitiveness of *Le*YC36 is multidimensional. Comparative transcriptome analysis showed that α-terpinene up-regulated the expression levels of exopolysaccharide synthesis genes (*LepslA*, *LepslD*, and *LepslE*) in *Le*YC36 (Fig. 2A). At present, the major exopolysaccharide components of *L. enzymogenes* have not been identified. Analysis results revealed partial conservation between the exopolysaccharide biosynthesis cluster in *Le*YC36 and the *psl* cluster of *Pseudomonas aeruginosa* (Fig. 3B). Four genes exhibiting high sequence similarity to *psl* homologs were designated *LepslA*, *LepslD*, *LepslE*, and *LepslI* (Table S1). We first identified whether these three genes, *LepslA*, *LepslD,* and *LepslE*, are involved in the biofilm biosynthesis of *Le*YC36. We deleted the *pslE* gene to obtain mutant *Le*Δ*pslE*. The mutants *Le*Δ*pslA* and *Le*Δ*pslD* strains we used were provided by our laboratory. The results showed that the biofilm production of the three mutants was inhibited, and the promotion effect of α-terpinene on biofilm formation was lost (Fig. 3C). This suggests that *LepslA*, *LepslD*, and *LepslE* are key factors to *Le*YC36 biofilm formation. The qPCR results were consistent with the comparative transcriptome results, and the expression levels of these three genes were significantly up-regulated by α-terpinene (Fig. 3D), suggesting that α-terpinene could promote biofilm formation by regulating the expression of biofilm synthetic genes.

### *Le*YC36 recognizes and responds to α-terpinene signals through the *Le*CzcS/*Le*CzcR system

**Fig 4:**
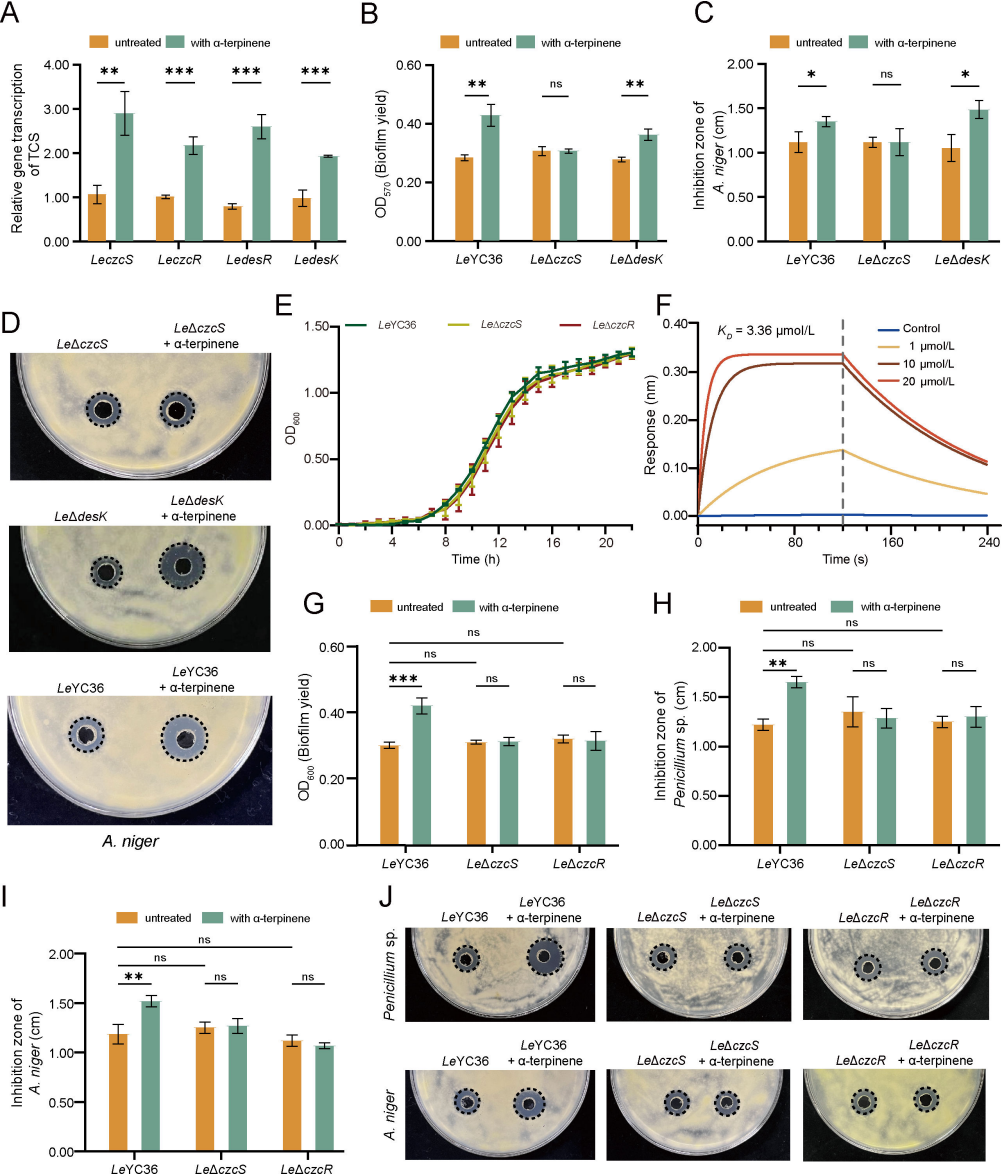
The *Le*CzcS/*Le*CzcR two-component system is involved in the α-terpinene-mediated signaling pathway to regulate antifungal activity of *Le*YC36. (**A**) qPCR results of *LeczcS*, *LepczcR, LedesR,* and *LedesK*. (**B**) Biofilm production assay of *Le*Δ*czcS* and *Le*Δ*desK*. (**C**) The inhibition zone diameter results of *Le*Δ*czcS* and *Le*Δ*desK*. (**D**) Agar diffusion assay of *Le*Δ*czcS and Le*Δ*desK* fermentation supernatant. The data for significance are shown in panel C. (**E**) Growth curves of *Le*Δ*czcS* and *Le*Δ*czcR*. (**F**) The binding (dissociation constant *K_D_*) between *Le*CzcS and α-terpinene. Control: *Le*DesK + 20 µM α-terpinene; *K_D_*: 3.36 M; R^2^: 0.9037. (**G**) Biofilm production assay of *Le*Δ*czcS* and *Le*Δ*czcR*. (H, I) The inhibition zone diameter results of *Le*Δ*czcS* and *Le*Δ*czcR*. (**J**) Agar diffusion assay of *Le*Δ*czcS* and *Le*Δ*czcR* fermentation supernatant. The data for significance are shown in panels **H and I**. Error bars show the standard deviation of three replicates. *P < 0.05, **P < 0.01, ***P < 0.001, ns is not significant. Data presented as mean  ±  SD.

**Fig 5:**
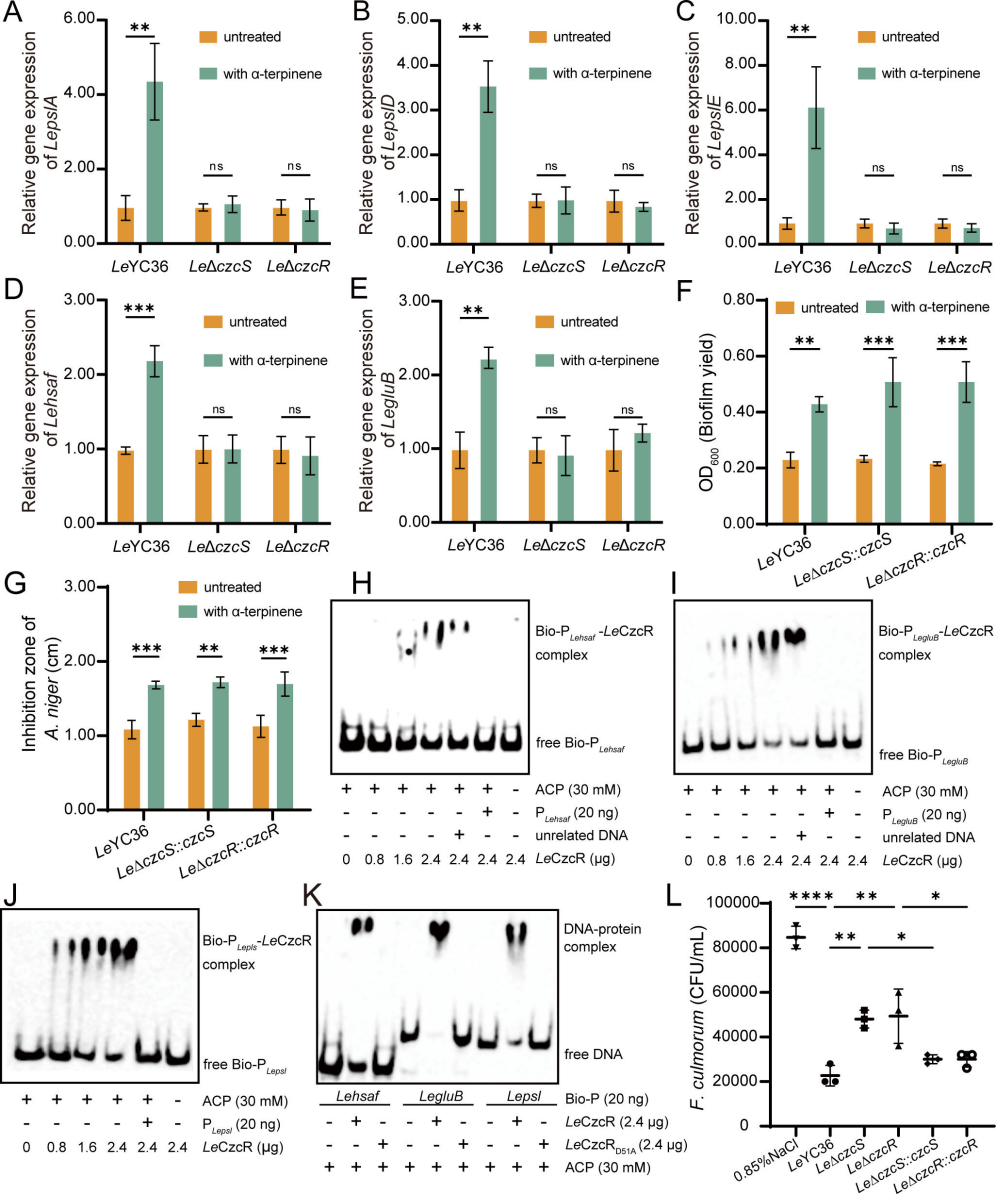
*Le*YC36 recognizes and responds to α-terpinene signaling molecule through *Le*CzcS/*Le*CzcR two-component system. (**A–E**) qPCR results of *LepslA*, *LepslD, LepslE, Lehsaf*, and *LegluB*. (**F**) Biofilm production assay of *Le*Δ*czcS::czcS* and *Le*Δ*czcR::czcR*. (**G**) The inhibition zone diameter results of *Le*Δ*czcS::czcS* and *Le*Δ*czcR::czcR*. (**H–J**) Binding of phosphorylated *Le*CzcR *in vitro* to the *Lepsl*, *Lehsaf*, and *LegluB* promoters. Each system contained 20 ng of biotinylated promoter DNA. Unrelated DNA was loaded at 40 ng. The primer sequences for the promoter and unrelated DNA are detailed in Table S2. The forward primer of the promoter carries a 5′-biotin modification. (**K**) Electrophoretic mobility shift assay (EMSA) results of *Le*CzcR_D51A_. Each reaction solution (10 µL) contained 30 mM acetyl phosphate (ACP). (**L**) CFU assay of *Fusarium culmorum* following bacterial-fungal co-culture. Error bars show the standard deviation of three replicates. *P < 0.05, **P < 0.01, ***P < 0.001, ****P < 0.0001, ns is not significant. Data presented as mean  ±  SD.

The results above confirmed that the signaling molecule α-terpinene can regulate the antifungal effectors and biofilm synthesis-related genes in *L. enzymogenes*. Then we would explore how *Le*YC36 recognizes and responds to the α-terpinene signaling molecule in the environment. Bacteria sense external signals and respond to environmental changes via two-component systems (TCSs), consisting of a sensor kinase and response regulator. Transcriptomic analysis revealed that α-terpinene significantly up-regulated the expression of two regulators, *Le*CzcR and *Le*DesR (Fig. 2A). Gene expression analysis of the two TCSs confirmed the transcriptomic results (Fig. 4A). We then deleted the genes of both TCSs and conducted biofilm production assays and antifungal assays on the mutant strains. The results showed that the α-terpinene-induced increase in antifungal activity and biofilm formation disappeared in the mutant *Le*Δ*czcS*. However, in the absence of gene *LedesK*, α-terpinene still induced an increase in the two physiological phenomena (Fig. 4B through D). Growth curves demonstrated that the deletion of *LeCzcS* or *LeCzcR* did not affect the growth ability of *Le*YC36 (Fig. 4E), indicating that the observed physiological phenomena were not due to growth inhibition. Concurrently, bio-layer interferometry (BLI) revealed that *Le*CzcS (see Fig. S1 for purified protein and sequence) binds directly to α-terpinene *in vitro* in a concentration-dependent manner, whereas no such interaction was detected with *Le*DesK (Fig. 4F). These results suggest that *Le*CzcS is involved in α-terpinene recognition and that the *Le*CzcS/*Le*CzcR system plays a role in the α-terpinene signaling pathway in *Le*YC36. Physiological experiments showed that the α-terpinene-induced increase in biofilm formation was abolished in the regulator mutant *Le*Δ*czcR* (Fig. 4G), and the enhanced antifungal activity was similarly lost (Fig. 4H through J). The qPCR results were consistent with the physiological observations, showing that the gene expression levels of biofilm and antifungal effector synthesis in the mutant *Le*Δ*czcR* remained unchanged in α-terpinene conditions (Fig. 5A through E). HSAF production in the *Le*Δ*czcS* and *Le*Δ*czcR* mutants was no longer enhanced by α-terpinene (Fig. S2), consistent with the qPCR results for the *Lehsaf* gene in the TCS mutants. To further confirm the role of the *Le*CzcS/*Le*CzcR system, we constructed gene complementation mutants *Le*Δ*czcS::czcS* and *Le*Δ*czcR::czcR*. Compared with wild-type and deletion mutants (Fig. 4G and H), both complementation strains regained responsiveness to α-terpinene, showing significantly increased biofilm formation and antifungal activity (Fig. 5F and G). These results validate the regulatory role of *Le*CzcS/*Le*CzcR in the α-terpinene signaling pathway in controlling downstream functional genes.

To further investigate whether *Le*CzcR (Fig. S3) directly regulates these downstream genes, we performed an electrophoretic mobility shift assay (EMSA). The results demonstrate that phosphorylated *Le*CzcR can bind to the promoter of *Lehsaf* (Fig. 5H), *LegluB* (Fig. 5I), and *Lepsl* (Fig. 5J), thereby directly regulating the expression of these genes. Through conserved sequence analysis based on homologous protein alignment (Fig. S4), the Asp51 residue of *Le*CzcR might be a key conserved phosphorylation site. Similar to unphosphorylated *Le*CzcR (Fig. 5H through J), the site-directed mutant *Le*CzcR_D51A_ (Fig. S5) failed to bind the promoter in EMSA assays (Fig. 5K). Together, these results identify Asp51 as the critical phosphorylation site in *Le*CzcR and demonstrate that phosphorylation is essential for promoter binding, thereby directly regulating downstream gene expression in the α-terpinene signaling pathway. *Fusarium culmorum*, a known α-terpinene-producing fungus (16), exhibited markedly inhibited growth when exposed to the *Le*YC36 fermentation supernatant (Fig. S6). In co-culture assays, *F. culmorum* showed significantly higher survival rates when paired with the signal transduction mutants *Le*ΔczcS and *Le*ΔczcR, whereas survival was reduced in the presence of the complementation strains, similar to the wild-type strain (Fig. 5L). These findings indicate that sensing fungal-derived α-terpinene through the *Le*CzcS/*Le*CzcR signaling pathway enhances the antifungal activity of the bacterium during microbial interactions.

**Fig 6:**
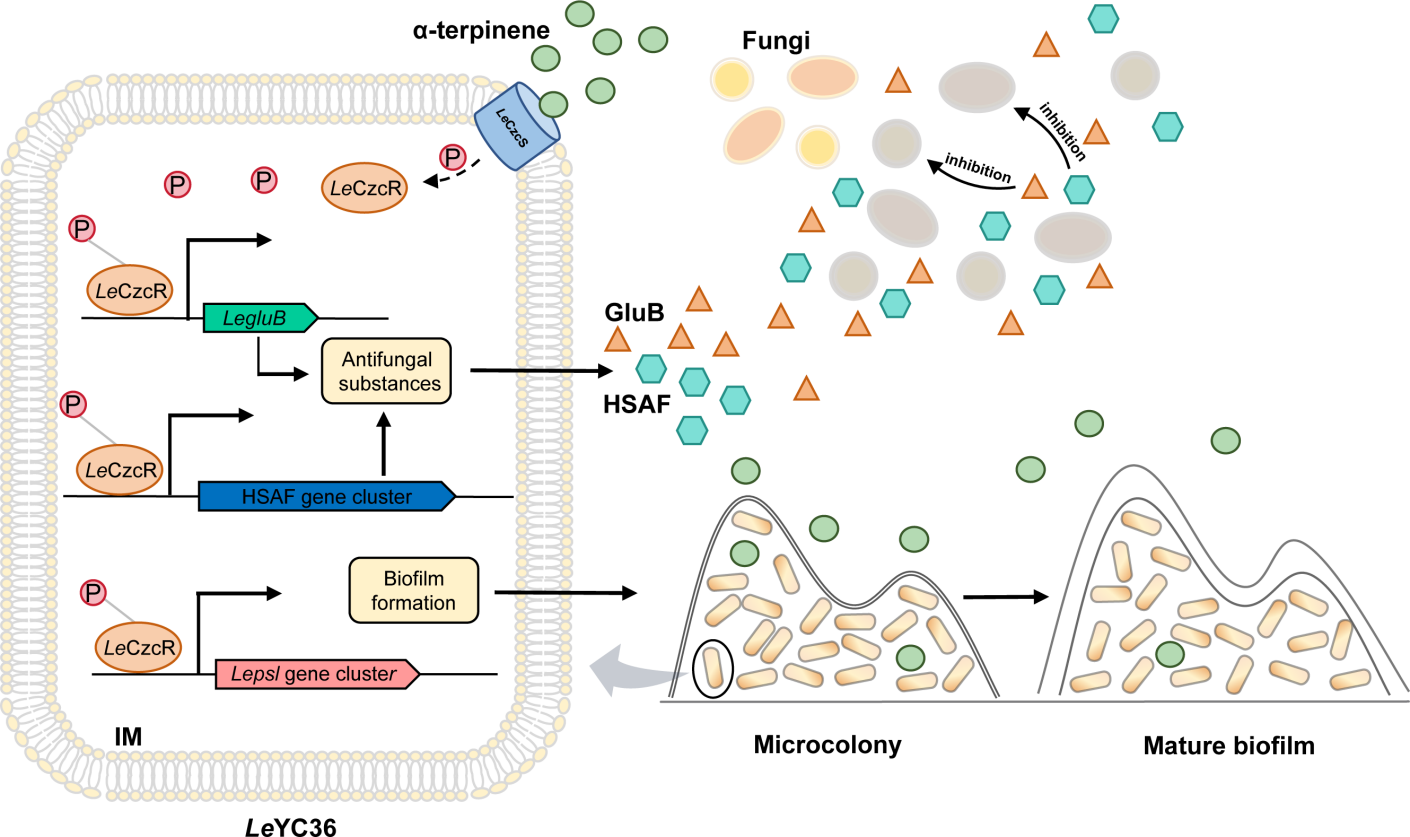
Mechanism diagram of α-terpinene-mediated signaling pathway in *Le*YC36 against fungi.

Based on the above results, it can be inferred that when *Le*CzcS detects the fungal signaling molecule α-terpinene, it transduces the signaling information to *Le*CzcR. *Le*CzcR will be activated by phosphorylation. Activated *Le*CzcR then binds to the promoters of target genes, promoting the expression of downstream genes associated with biofilm formation and antifungal effectors synthesis. Ultimately, this signaling cascade enhances the competitiveness of *Le*YC36 in interactions with fungi (Fig. 6).

## DISCUSSION

Microorganisms are ubiquitously present in diverse environmental communities, where their close physical associations form the foundation for critical interactions within ecosystems (28). These interactions are typically driven by chemical communication between different microbial communities, with the transfer of chemical signaling molecules between fungi and bacteria being particularly crucial (1, 4, 29, 30). However, current research on cross-kingdom interactions between bacteria and fungi mainly focuses on the effects of bacteria on fungal growth, metabolite production, and quorum sensing (8, 31, 32). In contrast, research on the regulatory effects of chemical signals produced by fungi on bacteria remains limited, with the focus primarily on bacterial quorum sensing and the inhibition of bacterial growth (33–36). For example, the metabolite farnesol produced by *Candida albicans* can regulate the expression of virulence genes in *P. aeruginosa* by interfering with bacterial quorum sensing (37). Additionally, Rasmussen et al. (38) have shown that *Penicillium* can secrete quorum-sensing inhibitors, thereby exerting a significant influence on bacterial behavior. These findings suggest that fungal chemical signals play a crucial role in BFI and may profoundly influence bacterial ecological behaviors and functions by regulating quorum-sensing mechanisms.

However, the specific regulatory mechanisms and ecological significance of these signaling molecules require further investigation. In particular, the functions and mechanisms of terpenes secreted by fungi as signaling molecules in BFI have not been fully elucidated. Current research has primarily focused on the effects of fungal terpenes on bacterial motility. For instance, Schmidt et al. (16) demonstrated that fungal terpene compounds can influence bacterial swimming and swarming. This study focused on the fungal terpene α-terpinene as a signaling molecule and examined its effects and mechanisms in regulating antifungal activity and biofilm formation in the environmental bacterium *Le*YC36. The findings offer new insights into microbial signaling and functional cooperation, contributing to a deeper understanding of microbial communication.

This study reveals the molecular mechanism through which α-terpinene enhances the antifungal capacity of *L. enzymogenes*, initiated by the *Le*CzcS/*Le*CzcR signaling pathway. Two key antifungal effectors, HSAF and GluB, are involved in this process. When the synthesis of HSAF and GluB is inhibited, the promoting effect of α-terpinene on the competitiveness of *Le*YC36 is abolished (Fig. 2C and G). Furthermore, the results of the inhibition zone assay showed that the mutant *Le*Δ*hsaf* exhibited almost no inhibition of fungal growth (Fig. 2G), whereas the mutant *Le*Δ*gluB* still retained its ability to inhibit fungal formation (Fig. 2C). This indicates that during the interaction between *Le*YC36 and fungi, HSAF is central to suppressing fungal growth. Given that HSAF targets the biosynthesis of sphingolipid in fungal cell membranes to disrupt the polarized growth of fungi (39), the significant upregulated expression of the hydrolase GluB in *Le*YC36 responding to α-terpinene (Fig. 2A and B) likely accelerates the degradation of fungal cell walls. This, in turn, enhances the targeting effect of HSAF on fungal cell membrane formation, generating a synergistic antifungal effect. This synergistic defense strategy allows *L. enzymogenes* to dynamically adjust its antimicrobial resource allocation in response to environmental signals, maximizing defense efficiency against fungal competitors. When *Le*YC36 detects fungal signals, α-terpinene in the environment, it recognizes the presence of competitors. In response, *Le*YC36 boosts its antifungal capabilities while increasing the production of biofilms, thereby strengthening its offensive and defensive mechanisms. In addition to the natural defense functions, biofilm also plays an active role in promoting the antifungal capabilities of *Le*YC36. It has been reported that biofilms contribute to the colonization and growth of *Lysobacter* strains on plant roots (40). The biofilms of biocontrol bacteria can form exclusion zones that prevent the colonization of pathogenic microorganisms (41). α-Terpinene-induced biofilm formation in *Le*YC36 likely enhances its colonization of the plant rhizosphere, establishing an exclusion zone that limits the invasion and infection of pathogenic fungi. This dual-regulation mechanism on antifungal effectors and biofilms provides a novel perspective for understanding the ecological adaptability of biocontrol bacteria. And our work will contribute to the further applications of biocontrol bacteria *Le*YC36 against pathogenic fungi.

We have identified the *Le*CzcS/*Le*CzcR system as playing a pivotal role in sensing α-terpinene signal and regulating downstream gene expression. However, its molecular mode of action differs significantly from that of the CzcS/CzcR system in *P. aeruginosa*. While the CzcS/CzcR system primarily regulates zinc homeostasis, quorum sensing, and antibiotic resistance in *P. aeruginosa* (42, 43), our study suggests that this system has evolved a novel function for chemical signal recognition in *L. enzymogenes*. Nevertheless, the structural determinants of α-terpinene recognition in *Le*CzcS await characterization. Further investigation of the specific interaction mechanism between the periplasmic domain of *Le*CzcS and α-terpinene would significantly advance our understanding of microbial cross-kingdom signal transduction networks. Notably, the α-terpinene biosynthetic pathway in fungi remains uncharacterized. To investigate its functional role, we co-cultured *L. enzymogenes* wild-type and TCS-deletion mutant strains with the α-terpinene-producing fungus *F. culmorum* (Fig. 5L). The results further confirmed that recognition of fungal-derived α-terpinene enhances the antifungal activity of *Le*YC36. Future studies should employ targeted gene knockout in *F. culmorum* to elucidate the complete regulatory role of α-terpinene in *Lysobacter*-fungal interactions.

## MATERIALS AND METHODS

### Bacterial and fungal strains, growth conditions, and materials

*A. niger* ATCC 1640, *Penicillium* sp., and *F. culmorum* CGMCC 3.4283 were cultivated at 28°C in potato dextrose agar (PDA) medium. *Le*YC36 and its mutant strains were grown at 28°C in 40% trypticase soy broth (TSB). *Escherichia coli* strains DH5α (Vazyme, C502-02), BL21 (DE3) (Vazyme, C504-02), and S17-1 λpir (AngYuBio, G6055) were utilized for DNA manipulation. All molecular manipulations in this study were performed according to established methods (44). The strains and plasmids used are listed in Table S1. Reagents required for molecular biology and biochemistry were purchased from Takara (Japan) and Yeasen (China). PCR primers were synthesized by Sangon Biotech (China) and Beijing Tsingke (China), along with sequencing services, which were provided by these companies (Table S2). Unless otherwise stated, the concentration of α-terpinene (picasso-e.com) used in the experiments was 0.1 μM, and the solvent of α-terpinene was dimethyl sulfoxide (DMSO). In the control group, DMSO was added at a volume equivalent to that of α-terpinene in the experimental group.

### Biofilm formation assay

To determine the effect of signal molecules on biofilm formation, *Le*YC36 was inoculated at 1% (vol/vol) into 40% TSB medium and cultured at 28°C, 200 rpm until the logarithmic growth phase (OD_600_ = 0.6–0.8). Bacterial cultures were diluted to an OD_600_ of 0.045, as measured using a microplate reader (Thermo Fisher Scientific, America). In the experimental group, the 0.1 µM α-terpinene was added. A total of 200 µL of each adjusted culture was transferred into 96-well plates, with three biological replicates for each group. Plates were placed at 28°C for 2–3 days. After incubation, culture media were gently discarded, and wells were washed thrice with 100 µL sterile ultrapure water. Plates were air-dried at room temperature. Biofilm was fixed with 100 µL methanol for 20 min, followed by staining with 100 µL of 1% crystal violet for 20 min. Excess stain was washed off with sterile ultrapure water (three washes), and the plates were air-dried. Ethanol (95%, 80 µL) was added to each well to solubilize the stain, and absorbance at 570 nm was measured using the microplate reader.

### Fungal survival rate (CFU assay)

#### Preparation of spore suspensions

*A. niger*, *Penicillium* sp., and *F. culmorum* were activated and cultured on PDA medium at 28°C for 3 days. Spores were suspended in sterile saline, vortexed, and filtered through sterile cotton to remove hyphae. The suspension was homogenized, and the concentration was adjusted to the desired level using a hemocytometer. For the CFU assay, activated *Le*YC36 cultures (1% inoculation in 40% TSB) were treated with signaling molecules and grown at 28°C, 200 rpm, until the logarithmic phase. The *Le*YC36 culture and spore suspension were mixed in a 2:1 vol ratio and co-incubated at 28°C for 24 h. In the experimental group, α-terpinene was added. Then, the co-culture suspension was serially diluted and spread onto PDA medium, and fungal colonies were counted after 1–2 days of incubation.

### Agar diffusion assay

Activated *Le*YC36 was inoculated at 1% (vol/vol) into 40% TSB medium with (experimental group) or without (control group) signal molecules and fermented at 28°C, 200 rpm for 2–3 days. After fermentation, 1 mL of the culture was centrifuged (5,000 rpm, 6 min), and the supernatant was collected. Spore suspensions (adjusted to 10^5^ CFU/mL) were spread onto PDA plates. Wells were punched into the agar using a 200 µL pipette tip, and 20 µL of the supernatant was added to each well. Plates were incubated at 28°C for 1–2 days, and the diameter of inhibition zones was measured using the vernier caliper.

### Growth curve assay

Activated *Le*YC36 and its mutants were inoculated at 1% (vol/vol) into 40% TSB liquid medium and cultured at 28°C, 200 rpm until the logarithmic growth phase. Adjust the OD_600 nm_ of each strain to 0.045, and transfer to fresh 40% TSB liquid culture medium (without antibiotics) at a 1% (vol/vol) inoculum volume. If special treatment is required, add corresponding reagents (such as 0.1 µM α-terpinene or DMSO). A 300 µL of the inoculated medium was added to a microplate. Growth curves were measured using Bioscreen C° Pro (Lab Systems, Finland). Cultures were incubated at 28°C, with OD_600_ measurements taken hourly over 24 h. Each sample was cultured in triplicate.

### Construction of gene deletion and complementation mutants

Gene deletion mutant strains of *L. enzymogenes* were constructed as described previously (44, 45). Using the *Le*YC36 genome as a template, the upstream and downstream homologous arm fragments of the target gene were obtained via PCR. The amplified fragment was digested with restriction enzymes along with the pEX18Gm plasmid, and the ligation reaction was used to construct a recombinant plasmid, which was then transformed into *E. coli* DH5α for screening and PCR validation. The validated recombinant plasmid was transferred to *E. coli* S17-1 competent cells and conjugated with *Le*YC36. Conjugation products were subjected to antibiotic selection to obtain strains with integrated recombinant constructs. Sucrose plates were used to remove strains carrying the suicide vector, and the successful deletion mutants were verified by PCR. Gene complementation strains were generated following the same strategy: target genes flanked by homologous arms were PCR-amplified and ligated into pEX18Gm to construct recombinant vectors. These were then introduced into corresponding deletion mutants via conjugation. The validated mutants were stored at −80°C for subsequent experiments.

### Gene transcription detection

The activated *Le*YC36 was inoculated at 1% into 40% TSB medium with 0.1 µM α-terpinene (experimental group) or without α-terpinene (control group) and incubated at 28°C, 200 rpm. Thirty minutes before reaching the logarithmic phase, the experimental group was supplemented with α-terpinene to a final concentration of 0.1 µM and cultured to the logarithmic phase. Bacteria were collected, and RNA was extracted using the Bacterial RNA Kit (Yeasen, China) following the manufacturer’s protocol. The RNA concentration was measured with the micro-spectrophotometer (Thermo Fisher Scientific, America), reverse-transcribed into cDNA using the Reverse Transcription Kit (ABM, Canada), and stored at −20°C. Real-time PCR was performed in a total reaction volume of 20 µL with Hieff qPCR SYBR Green Master Mix (Yeasen, China) and the StepOne Real-time PCR System (Applied Biosystems, America), and 16S rRNA was used as the reference gene. All experiments were collected from three biological replicates.

### Transcriptional analysis

Analysis of the transcriptome of *Le*YC36 under 0.1 µM α-terpinene-treated and untreated conditions was performed at Scientsgene Company, Qingdao, China. Cell culture procedures were performed as described in the “Gene transcription detection” section. The RNA concentration was measured using a micro-spectrophotometer, followed by sequencing on the Illumina HiSeq platform. High-quality, clean data were obtained after quality control of the raw sequencing data. The clean reads were then aligned to the *Le*YC36 reference genome to generate mapping data. Significantly upregulated genes were defined based on the criteria of log2 (Fold Change) >0 and *P* value <0.05. These genes were annotated using the Kyoto Encyclopedia of Genes and Genomes database to predict their functions and associated metabolic pathways.

### Heterologous expression and purification

The DNA sequence of *LeczcR* was amplified using the *Le*YC36 genome as a template and ligated into the pET-28a (+) expression vector. The recombinant plasmid was transformed into *E. coli* BL21 (DE3) for protein expression. The transformed strain was cultured in LB medium containing 100 µg/mL kanamycin at 37°C until the OD_600_ reached 0.6, after which IPTG was added to a final concentration of 0.1 mM. The culture was incubated at 16°C for 16 h. The bacteria were collected by centrifugation (8,000 rpm, 10 min), resuspended in binding buffer (20 mM Tris-HCl, 0.5 M NaCl, pH 8.0), and disrupted using an ultrasonic cell disruptor (JY92-IIN, Scientz, China) three times (35% power, 10 min). After centrifugation at 4°C (12,000 rpm, 10 min), the supernatant was filtered through a 0.22 µm membrane, and the recombinant protein was purified using elution buffer (20 mM Tris-HCl, 0.5 M NaCl, 10/20/50/75/100/250/500 mM imidazole, pH 8.0). To remove phosphate groups and imidazole from the protein, the sample was first dialyzed once against an acidic buffer A (20 mM Tris-HCl, 100 mM KCl, 50 mM NaCl, 2 mM EDTA, 10% glycerol, 0.5% Tween 20, pH 5.0–5.5), followed by four changes of neutral buffer B (modified from buffer A, pH 7.0–7.5). Protein purification was verified by sodium dodecyl sulfate-polyacrylamide gel electrophoresis, and the protein concentration was determined using the Bradford method with a commercial kit.

For site-directed mutation, Clustal W in MEGA was used to align the protein sequences belonging to the same class as *Le*CzcR, and ESPript 3.0 was used for analysis to obtain the conserved regions. The recombinant plasmid *Le*CzcR_D51A_-pET-28a (+) was amplified from *Le*CzcR-pET-28a (+) with site-directed mutation primers (Table S2) and repaired by *E. coli* DH5α. Then, this mutant plasmid was transferred into *E. coli* BL21 (DE3), and the purification of the mutant protein followed the above method.

### Extraction and HPLC analysis of HSAF

Wild-type and mutant strains were grown in 40% TSB medium with or without α-terpinene for 2 days. After centrifugation, the supernatant (3 mL) was acidified to pH 3.5 with concentrated HCl. After adding 0.5 g CaCl_2_ and complete dissolution, 3 mL ethyl acetate was introduced. The mixture was vortex-mixed (2,000 rpm, 1 min), then centrifuged (8,000 rpm, 5 min). The organic phase was collected, evaporated, and the HSAF-containing residue was reconstituted in methanol. Following 0.22 µm filtration, the clarified extract was analyzed by high-performance liquid chromatography (HPLC) (46). The HPLC condition was performed as described previously (47).

### The binding between *Le*CzcS and α-terpinene *in vitro*

*In vitro* binding analysis was performed via BLI. Purification of membrane protein *L*eCzcS and *L*eDesK was performed as described previously (44), and the vector used for heterologous expression of membrane protein is pET-19b. Purified membrane protein was dialyzed in PBST (PBS + 0.02% Tween-20) and later loaded onto Ni-NTA biosensors (Sartorius, Germany). Concentration-dependent binding to α-terpinene (1, 10, 20 µM) was quantified on the Octet R8 System (Sartorius, Germany). Membrane protein *L*eDesK was also loaded onto biosensors as a control.

### Electrophoretic mobility shift assay

Based on the method described by Liu et al. (48), some steps were modified. The purification of *Le*CzcR was performed as described above. The promoter sequence was predicted using promoter prediction tools (BDGP: Neural Network Promoter Prediction), and specific primers were designed. Using the genome of *Le*YC36 as a template, DNA fragments of the promoter region were amplified. The fragments were purified using a Gel/PCR Purification Kit and diluted to a final concentration of 50 ng/µL. The EMSA reaction system (10 µL) contained 20 ng of biotin-DNA, 20 ng of unlabeled DNA as competitors, 40 ng of unrelated DNA from the *Le*YC36 genome as a control, 30 mM acetyl phosphate (ACP), 2.4 µg of *Le*CzcR_D51A_ as the protein control, and different concentrations of *Le*CzcR. The reaction mixture was incubated at 25°C for 30 min, and the samples were separated using a 6% native polyacrylamide gel at a constant voltage of 110 V for 90 min. Then, the compounds were transferred to the nylon membrane (Millipore, USA) at 350 mA for 90 min. Streptavidin-horseradish peroxidase conjugate was used to monitor the migration of biotinylated probes.

### Statistical analysis

Statistical significance was determined using Student’s *t*-test for comparisons between two groups and one-way analysis of variance for comparisons among multiple groups. Data are presented as mean ± SD (*n* = 3 biological replicates).

## SUPPLEMENTAL MATERIAL

Supplemental material aem.01471-25-s0001.pdf Figures S1 to S6; Tables S1 to S3.

ASM does not own the copyrights to Supplemental Material that may be linked to, or accessed through, an article. The authors have granted ASM a non-exclusive, world-wide license to publish the Supplemental Material files. Please contact the corresponding author directly for reuse.
