# Supplementary material for: Mechanism of Lysobacter enzymogenes resistance toward fungi induced by fungal-derived signal α-terpinene
Source: Appl Environ Microbiol. 2025 Sep 24;91(10):e01471-25. doi: 10.1128/aem.01471-25 (PMC12542728; doi:10.1128/aem.01471-25)
Supplement: Supplemental material — Figures S1 to S6; Tables S1 to S3. [file aem.01471-25-s0001.pdf]

The mechanism of *Lysobacter enzymogenes* resistance toward fungi induced by fungal-derived signal α-terpinene

Meixue Zhu[1], Yuying Li[1,2], Fang Nan[1], Jia Wang[1], Benfeng Gao[1], Huihui Song[1], Zeran Bian[1], Xuejie Wang[1], Yuxiang Zhu[1], Yan Wang[1,2,3]#

1. Supplemental figure

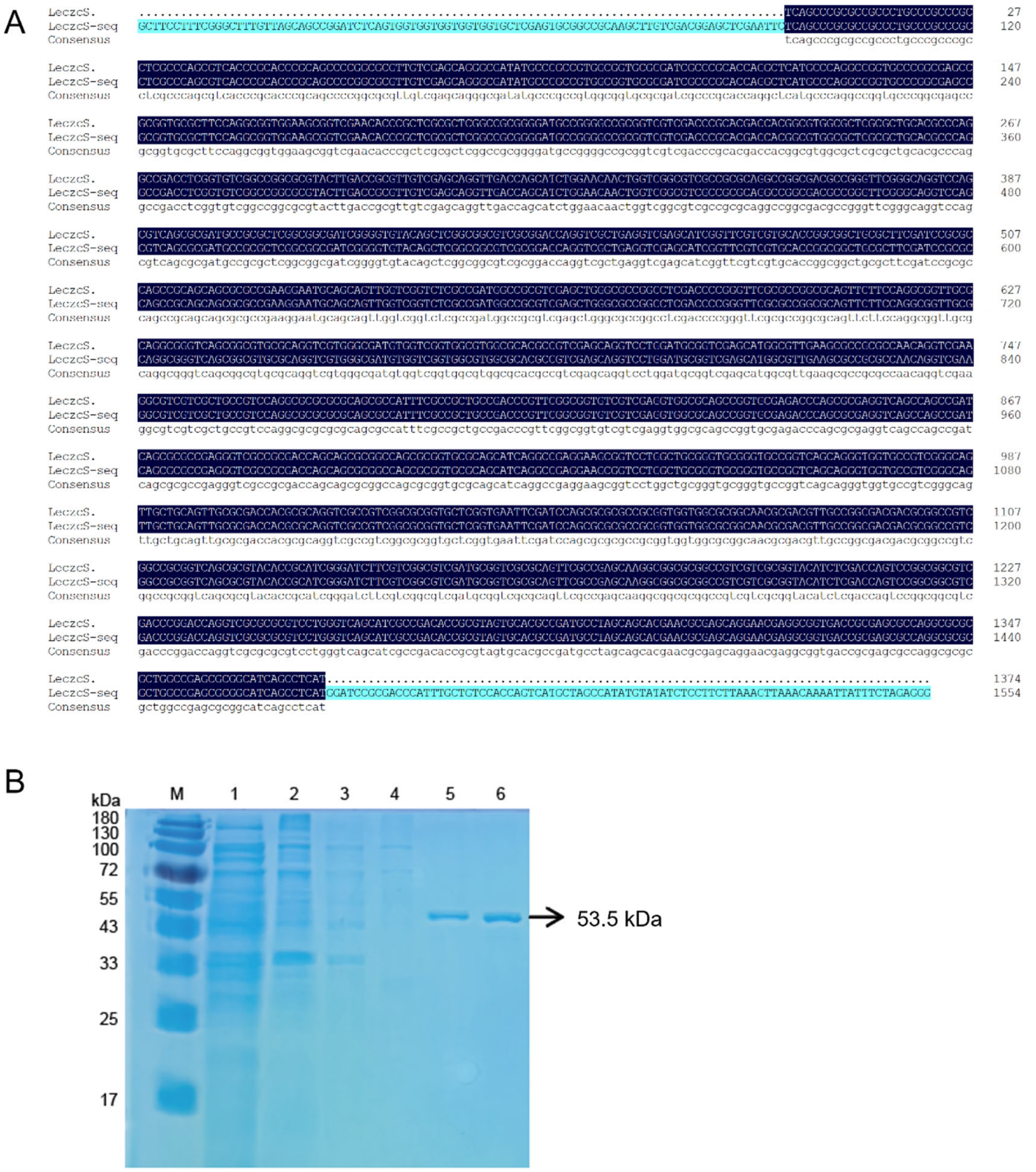

Fig. S1. The purified *Le*CzcS. (A). The sequencing result of the constructed

heterologous expression vector for *Le*CzcS. **(B).** SDS-PAGE analysis of purified *Le*CzcS. M: Protein marker; 1: *E. coli* BL21; 2–6: 10 mM, 50 mM, 100 mM, 250 mM, 500 mM imidazole elute. Optimal imidazole elution concentrations for *Le*CzcS were 250–500 mM.

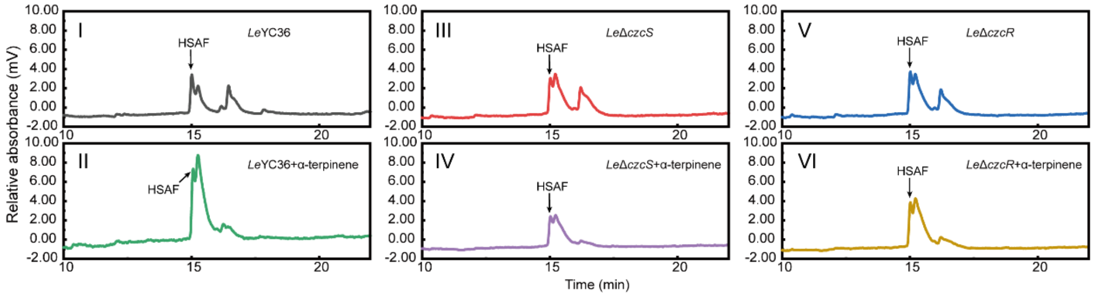

Fig. S2. HPLC results of HSAF produced by strains *Le*YC36, *Le*Δ*czcS*, and *Le*Δ*czcR* with or without α-terpinene.

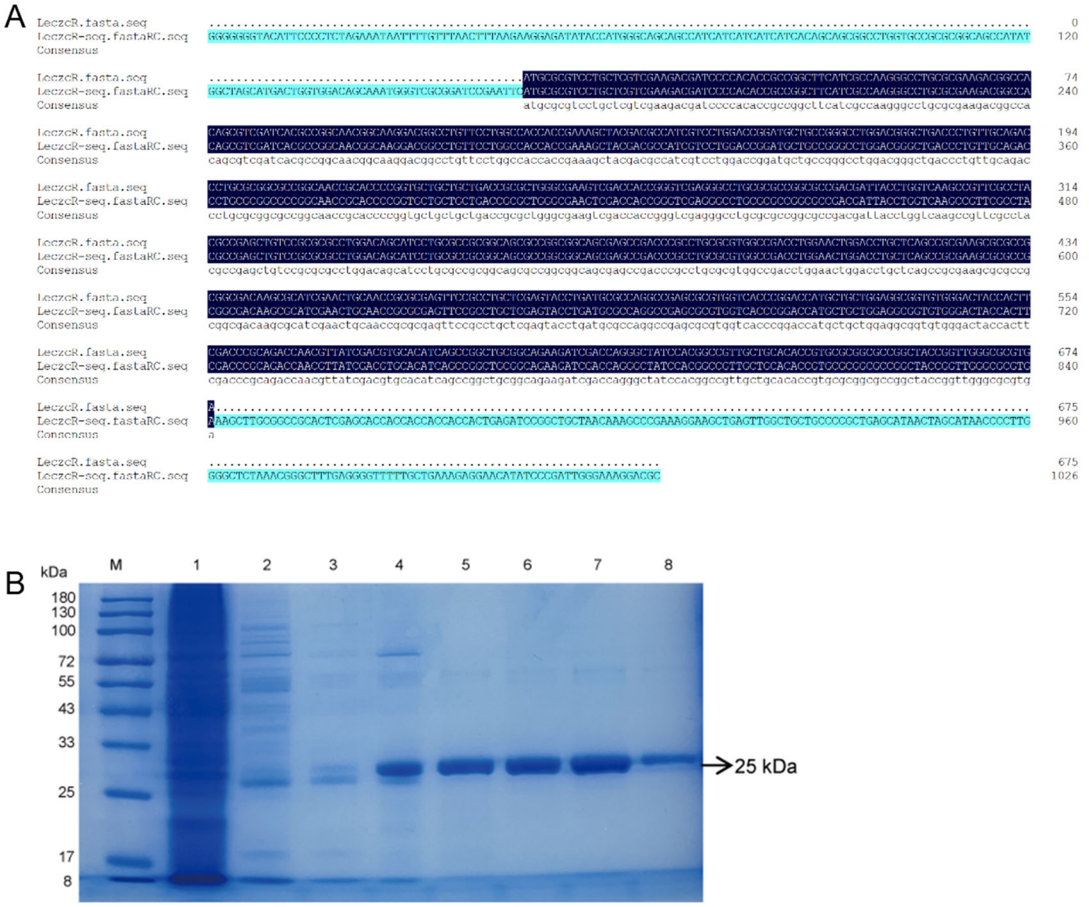

Fig. S3. The purified *Le*CzcR. (A). The sequencing result of the constructed heterologous expression vector for *Le*CzcR. **(B).** SDS-PAGE analysis of purified

*Le*CzcR. M: Protein Marker; 1: E. coli BL21; 2–8: 10 mM, 20 mM, 50 mM, 75 mM, 100 mM, 250 mM, and 500 mM imidazole elute. Optimal imidazole elution concentrations for *Le*CzcS were 100–250 mM.

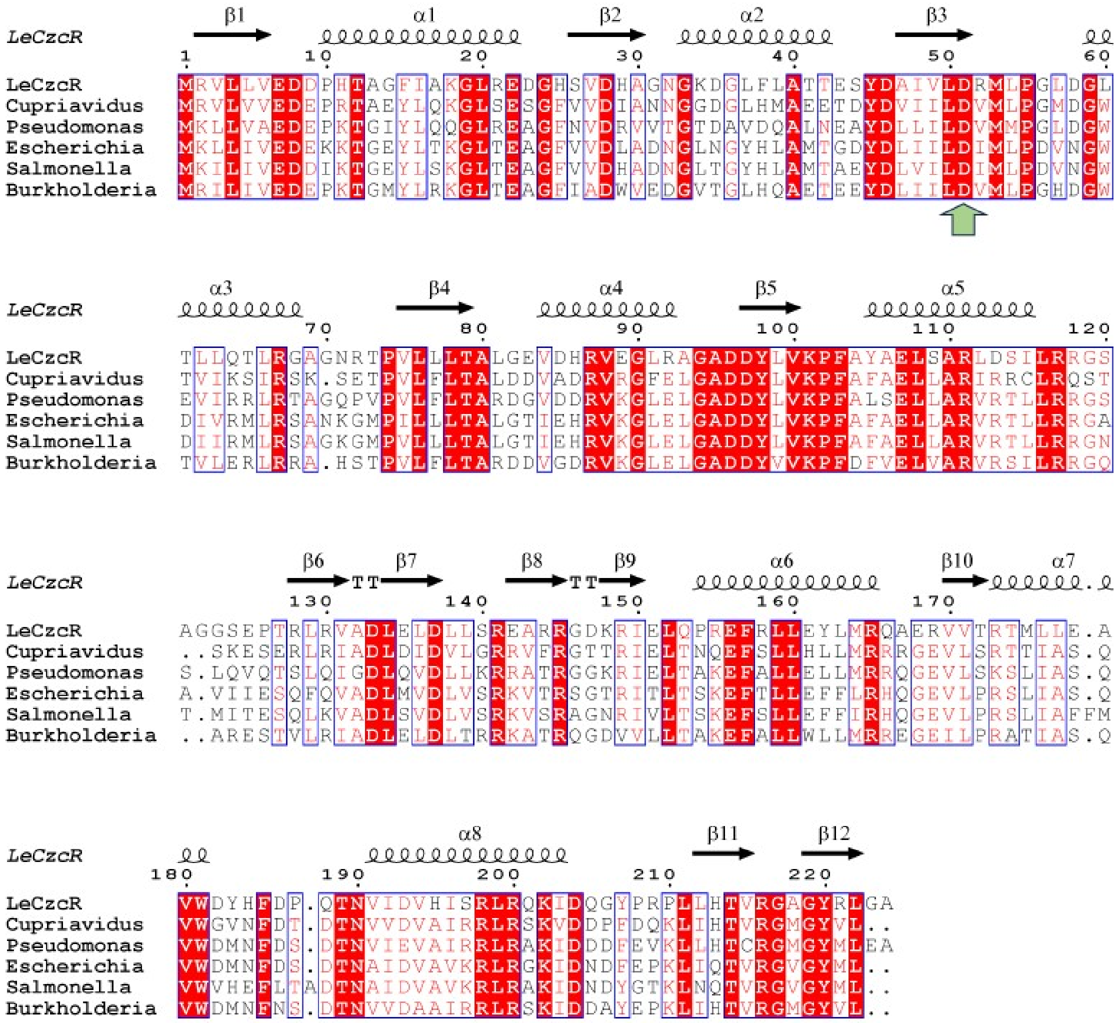

Fig. S4. Multiple sequence alignment of *Le*CzcR with homologous proteins. The consistency among amino acid sites were shown in red. The Asp51 residue of *Le*CzcR is a potentially conserved phosphorylation site, which is indicated by the green arrow.

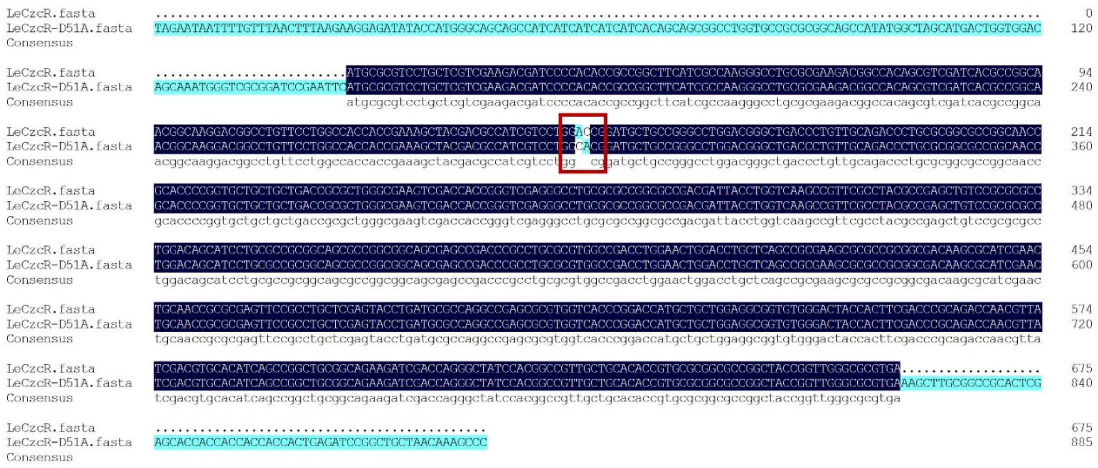

Fig. S5. The sequencing result of the constructed heterologous expression vector for *Le*CzcR$_{\mathbf{D51A}}$.

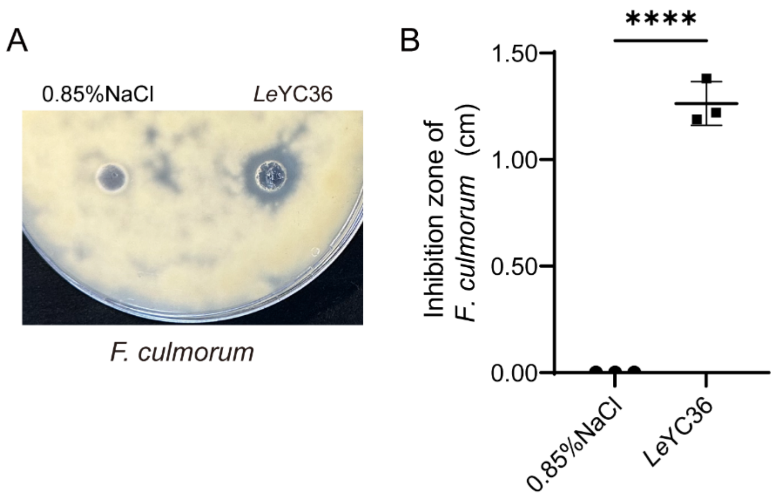

Fig. S6. *Le*YC36 fermentation supernatant inhibits the growth of *F. culmorum*. (A). Agar diffusion assay of *Le*YC36 fermentation supernatant to *F. culmorum*. The data for significance of panel A showed in panel B. **(B).** The inhibition zone diameter results. Error bars show the standard deviation of three replicates. **** $P < 0.0001$. Data presented as mean ± SD.

2. Supplemental tables

Table S1. The protein blast results for *Lepsl* and *psl* from *P. aeruginosa*.

***Le*YC36**	**Gene Function**	***P. aeruginosa* PAO1**	**Identity**	**Cover**
LepslA	Pellicle/biofilm biosynthesis protein PslA, polyprenyl glycosylphosphotransferase	*pslA*	57.45%	86%
LepslD	Polysaccharide biosynthesis/export protein, Sugar transporter	*pslD*	28.72%	72%
LepslE	Polysaccharide chain length determinant N-terminal domain-containing protein	*pslE*	22.68%	90%
LepslI	Pellicle/biofilm biosynthesis protein PslI, CAZy glycosyltransferase family 4	*pslI*	48.1%	98%

Table S2. The strains and plasmids.

**Strains**	**Relevant characteristics**	**Source**
Lysobacter enzymogenes YC36	Wild type strain	Lab storage
Penicillium sp.	Wild type strain	Lab storage
Aspergillus niger ATCC 1640	Wild type strain	Lab storage
Fusarium culmorum CGMCC 3.4283	Wild type strain, isolated from the soil	Lab storage
Escherichia coli DH5α	Competent cells that can be used for DNA manipulation	Lab storage
Escherichia coli BL21 (DE3)	The strain used for heterologous expression of protein	Lab storage
Escherichia coli S17-1	The strain used as bacterial conjugal donor	Lab storage
*Le*Δ*pslA*	*LepslA* gene deletion mutant strain, in-frame deletion by pEX18Gm	Lab storage
*Le*Δ*pslD*	*LepslD* gene deletion mutant strain, in-frame deletion by pEX18Gm	Lab storage
*Le*Δ*pslE*	*LepslE* gene deletion mutant strain	This study
*Le*Δ*gluB*	*LegluB* gene deletion mutant strain, in-frame deletion by pEX18Gm	Lab storage
*Le*Δ*hsaf*	*Lepks/nrps*$_{\text{HSAF}}$ gene deletion mutant strain, in-frame deletion by pEX18Gm	Lab storage
*Le*Δ*czcS*	*LeczcS* gene deletion mutant strain	This study

*Le*Δ*czcR*	*LeczcR* gene deletion mutant strain	This study
*Le*Δ*desK*	*LedesK* gene deletion mutant strain	This study
BL21-*Le*CzcS	The heterologous expression strain of *Le*CzcS	This study
BL21-*Le*CzcR	The heterologous expression strain of *Le*CzcR	This study
BL21-*Le*CzcR$_{D51A}$	The heterologous expression strain of *Le*CzcR$_{D51A}$	This study
BL21-*Le*DesK	The heterologous expression strain of *Le*DesK	This study
*Le*Δ*czsS*::*czsS*	*LeczcS*-complemented strain	This study
*Le*Δ*czsR*::*czsR*	*LeczcR*-complemented strain	This study
**Plasmids**	**Relevant characteristics**	**Source**
pEX18Gm	The vector used for in-frame deletion and gene complement	Lab storage
pET28a (+)	The vector used for heterologous expression	Lab storage
pET-19b	The vector used for heterologous expression of membrane protein	Lab storage

Table S3. The primers.

**Primers**	**Sequence**	**Purpose**
LepslE-up-F-Hind III	cccaagcttcggcgacgagctcgcgctcaag	For gene in-frame deletion
LepslE-up-R	gcctggctgaggaatgtcgcggaaggcgatct	
LepslE-down-F	tcgccttccgcgacattcctcagccaggcgct	
LepslE-down-R-BamH I	cgcggatccccttccaggctgccgcccttgc	
LeczcS-up-F-Hind III	cccaagcttggcgccggcaaccgcaccc	
LeczcS-up-R	gcggtgcgcgattagtgcacgccgatgc	
LeczcS-down-F	tcggcgtgcactatcgcgcaccgccac	
LeczcS-down-R-BamH I	cgcggatccccgccgcctcgccgccac	
LeczcR-up-F-Hind III	cccaagcttttcatccgccggaaaggcc	
LeczcR-up-R	cacgtcgataactgccgttgccggcgtga	

LeczcR-down-F	cgccggcaacggcagttatcgacgtgcac	
LeczcR-down-R-BamH I	cgcggatcccgcagcatcaggccgaggaa	
LedesK-up-F-Hind III	cccaagcttagtggcggcgagcatcggcccc	
LedesK-up-R	tcgatttccagcacgatcgccagggccagga	
LedesK-down-F	tggccctggcgatcgtgctggaaatcgaatc	
LedesK-down-R-EcoR I	ccggaattcttacagccagccgttctggcgcg	
Lepsl-EMSA-F	tggtaggggcggattgc	EMSA
Lepsl-EMSA-R	ggccgggtgagttttgg	
LegluB-EMSA-F	gggtcgccgatgatcg	
LegluB-EMSA-R	acgggccgcaccttg	
Lehsaf-EMSA-F	attccaaagaatgatccgcgtcg	
Lehsaf-EMSA-R	tggtggtggtcggcccg	
LedesK-F-19b	atatcgacgacgacgacaagcaatgcgcacgcccggcccacc	Recombinant protein purification and site-directed mutation
LedesK-R-19b	tttcgggctttgttagcagccgttacagccagccgttctggc	
LeczcS-F-Nde I	ggaattccatatgatgaggctgatgccgc	
LeczcS-R-BamH I	cgcggatcctcatcgtaaccgcgcggg	
LeczcR-F-EcoR I	ccggaattcatgcgcgtcctgctcg	
LeczcR-R-Hind III	cccaagctttcacgcgcccaaccgg	
LeczcR$_{\mathrm{D51A}}$-F	ccatcgtcctggcacggatgctgccgggcc	
LeczcR$_{\mathrm{D51A}}$-R	agcatccgtgccaggacgatggcgtcgtagc	
16S-qPCR-F	gacgtcatcgtcagcaatcc	For real-time PCR
16S-qPCR-R	gttggcgaccatccagaaac	
LepslA-qPCR-F	gtgatgaccctgcacctact	
LepslA-qPCR-R	cacgaacaggacgaagctc	

LepslD-qPCR-F	caagtggatcgaccagtacg	
LepslD-qPCR-R	tgtcgttgaggtcgtaggag	
LepslE-qPCR-F	ccggctgtatcccgacatc	
LepslE-qPCR-R	aaaccgatctggatcacggt	
LegluB-qPCR-F	ctcgacccgatctgctactc	
LegluB-qPCR-R	tgtagccggtcaggtagttc	
Lehsaf-pks/nrps-qPCR-F	accttcatgattccctcgca	
Lehsaf-pks/nrps-qPCR-R	cagcaactgcttccagtgtt	
LeczcS-qPCR-F	cggactggtcgagatgtacc	
LeczcS-qPCR-R	tacaccgcatcgggatcttc	
LeczcR-qPCR-F	tgtgggactaccacttcgac	
LeczcR-qPCR-R	ggatagccctggtcgatctt	
LedesK-qPCR-F	ttgctctacctgagcttcgt	
LedesK-qPCR-R	agtacagcggcaggaaca	
LedesR-qPCR-F	cttctggccgaagaccaag	
LedesR-qPCR-R	atcacctcgatgtcgggttc	
